# Effect of cylinder-liner rotation on wear rate: An experimental study

**DOI:** 10.1016/j.heliyon.2019.e02065

**Published:** 2019-07-13

**Authors:** Sa'ed A. Musmar, Ammar Alrousan, Iskander Tlili

**Affiliations:** a Department of Industrial Engineering, School of Engineering, The University of Jordan, Amman 11942, Jordan; b Department of Industrial Engineering, Hijjawi Faculty for Engineering Technology, Yarmouk University. P.O. Box 566, Irbid 21163, Jordan; c Department for Management of Science and Technology Development, Ton Duc Thang University, Ho Chi Minh City, Viet nam; d Faculty of Applied Sciences, Ton Duc Thang University, Ho Chi Minh City, Viet nam

**Keywords:** Mechanical engineering, Industrial engineering, Materials science, Cylinder liner, Wear rate, Piston-rings, Angles

## Abstract

In rotating cylinder-piston system, the largest losses source is frictional losses, accounting for 50% of the total frictional losses, thus it is important to optimize. Effect of incremental rotation of a cylinder liner on its wear rate was investigated. The engine speed, load and the cylinder rotating angle were the main parameter. The results showed that the wear rate may be reduced to the half simply by rotating cylinder liner every six hours’ time interval of working. The test was carried out in pairs using a piston cylinder with movable liner and compared to a standard cylinder liner (fixed liner). Angles of 60^o^, 120^o^, 180^o^, 240^o^, and 300^o^ were used for incremental movement. The same operating conditions for two cylinders were maintained for the purpose of comparison. Beneficial effects of reducing the wear rate for all components of the piston-cylinder arrangement associated with incremental rotational movement of a cylinder linear were noticed. A decrease in wear rate was obvious in the cylinder liner in rotation angles of 120^o^ and 240^o^ and it is almost one-fourth of the wear that occurs in the stationary cylinder liner.

## 1 Introduction

Energy affects substantially our modern life which governed by fuel depletion. This compels researchers and automotive industry in addition to the high market competition toward enhancing engine performance and leads to significant thermal and mechanical tensions on piston and cylinder design.

Wear is taking place between any two solid surfaces in contact and have relative motion to each other; cylinder bore of an internal combustion engine is subjected to an excessive wear that may affect the engine performance and efficiency due to high relative velocity associated with high working temperature and pressure environment. Sealing function is strongly affected by lubrication scheme and wears condition, engine operating life will be affected. In such case, the key role would be the original design tolerances.

In last two decades, Engines technologies have been enhanced greatly. Maintaining high engines power and efficiency require regular maintenance actions. Improving engine parts has not been more critical especially for of high power. Modern design based on stronger and lighter materials, lesser clearances and enhanced designs are the key features of efficient engines. This leads to longer operating life. Moreover, reducing exhaust emission provoked tighter ring cylinder interface that makes wear issue even more critical [1].

In spite of improvements, there are still engines fail too hastily. This may be related to the operating conditions. Failure in the piston, ring cylinder areas is mainly due to excessive wear. This form of failure is indicated by high blow-by, high oil consumption, or both of them. When either reaches the too high level, the engine emissions increased significantly and the fuel consumption conspicuously increased. Both blow-by and oil consumption can be caused by a failure in some other part of the engine. The wear is not being level along the liner and this is due to the variable forces of the piston rings, the varying charge pressure and gas temperature in the cylinder. Once the power stroke stars, the combustion pressure forcing the piston rings to the liner surface is at its highest; the surface temperature of the linear is high (about 200 °C) and affecting lubricating oil in reducing its viscosity [2].

At this part of the liner length, the piston speed is low, and so is the oil is the only thin film. These are the conditions were high wear is anticipated and normally found at the top of a liner enlarges to a few centimeters below the position of the top ring at top dead center. Then the wear rate decreases as the piston moves toward the bottom dead center since the ring pressure and liner surface temperature decreases and the piston speed increases building up a hydrodynamic film between liner and ring surfaces. Wear is caused by mechanical rubbing of two surfaces causing tearing of small particulates due to applied shear stresses. When the surface material is weakened due to chemical corrosion, the piston ring wears at a considerably higher rate [2, 3].

There are indicating four possible wear patterns: normal wear, increased top-end wear, increased bottom-end wear and all wear rates increased. If corrective action is taken, the life of the component will be significantly increased, reducing maintenance costs. The surface contact problems between cylinders and pistons through diesel engine rings are vital to the engine performance, efficiency, and fuel consumption. The unfavorable operating conditions of high pressure, temperature rise, and high relative velocity of the contacting surfaces lead to high wear rate [4, 5, 6]. Z. Krzyzak et al. [7] examined experimentally the magnitude of the piston damage after changing piston skirts surfaces for the “zero-wear” situation. Through investigating piston skirts after engine functioning, they conclude that the impairment of piston surface has been reduced considerably. An original instrument to simulate the accurate friction between the cylinder liner and the piston rig independent of the speed value has been developed by Markus Söderfjäll et al. [8]. They found that a great effect on friction, results on running only the oil control ring about 1.3% then decreases to 0.6% with running all three rings and it declines to 0.3% in case of two top rings. It is well known that the contact cylinder liner - piston ring susceptible to friction and wear, mostly related to ring load and temperature, work period and sliding speed, nevertheless it is very hard to expect the physical effect of this parameter with realistic values, for this reason, Julian Biberger et al. [9] used rotational tribometer with a novel testing method to perform friction and wear at a different level of pressure combustion chamber, ring temperatures and sliding speeds, they observed that for a small load, friction declines intensely when increasing sliding speed and wear rises for lesser temperature, whereas it decreases with greater temperature. Enhancing the surface conditions with the fine finish and surface treatment together with tighter tolerances implementation are essential to ensure better lubrication conditions, less friction, low wear and consequently good sealing effect between the cylinder wall and piston rings that leads to high engine efficiency, and longer service life span [13, 14]. A tight dynamic sealing within combustion chamber may be achieved during high-pressure strokes, compression and power strokes with piston rings assembly. Which reduces friction force and prevent charge flee from the combustion through ring expansion gap and consequently minimizes power loss and enhances combustion efficiency. For enhancing service life of the sealing, friction and wear between piston rings and cylinder wall have to be minimized and control [15, 16]. Oil film associate with dry lubrication of cylinder bore material composition may be capable to control wear at piston rings and cylinder interface [5, 15].

The Improvement of the tribological performance and attempt against the friction of a piston ring and cylinder are the main objective of Zhinan ZHANG et al. [10]. They involve in their experimental study carbon nanotubes (CNTs) lubricant and lubricant additive, they report that the friction coefficient lessens as CNT augment. The study in [9] was further extended by B. Zabala et al. [11]. They carried out an experimental and a simulation investigation for wear of piston ring and cylinder liner depending on another parameter than that declared by [9]. Theses parameters are surface coatings and finishing, lubrication starvation and oil type. It is worthwhile to note that the DLC coatings generate less friction compared to other coatings. Jian Zhang et al. [12] conducted an experimental analysis to find the effect of manganese series phosphating (Mn-P) behavior on the friction coefficient and wear rate compared to other zinc series phosphating (Zn-P) treatment. They realize that for both higher pressure and poor lubrication manganese series phosphating treatment tend to lessen meaningfully friction of both factors and wear.

The friction fluctuates with piston velocity and so is the oil film thickness since it depends on the relative velocity of the piston ring, which varies from a maximum at the middle of the traveling distance of the position, between TDC and BDC, to a zero at the depart points. Accordingly, the wear rate will vary along the piston ring cylinder contacting surface, from mild to severe [17].

The cylinder liner “zero-wear” process using two-scale homogenized method has been explored by Xianghui Meng et al. [18] with simulation and experimental investigation, their model enhances and explores the period service of the cylinder liner and piston ring. The wear protection and the endurance of the tribofilm for piston ring and cylinder liner have been explored by S. Spiller et al. [19]. They reported that lower wear rate and friction coefficient established in case of carbon-rich tribofilm compared to tribofilms with extra basics additive. At higher temperatures, the alloying elements the Grey cast iron leads to enhance wear protection while is not chrome plated. N. Jayakumar et al. [20] compared the effect of chrome plated cylinder liner to the non-chrome plated cylinder; they realized that in case of chrome plated cylinder liner the life cylinder liner is amplified by 50%. An optimum scale for cylinder liner temperature leads to reduce friction loss and emission avowed by R. Rahmani et al. [21], they address numerically the impact of cylinder liner temperature on friction loss and energy performance by incorporating in their study thermodynamics and heat transfer with mechanical frictional losses.

In fact, the conformity of cylinder bore along its entire length is not possible. Practically, ovality of cylinder bore causes inhomogeneous oil film distribution and consequently uneven lubrication performance regimes [13, 15, 18] will be developed associated with different wear mechanisms that lead to geometrical departures in transverse sections along the cylinder bore [17]. Vicinity of the top dead center is hard to be lubricated and expected to experience rigorous wear also the vicinity of the bottom dead center may experience notable wear due to solid particles existence in the oil reservoir the splashes at that region due to the motion of the crack shaft and wear wreckage driven by gravity and accumulated at the end position of piston stroke (near bottom dead center).

To the best of author's apprehension, nobody has discussed the effect of rotating the cylinder liner on the wear in internal combustion engines at a different angle and engine speed. In this work, a simple and specific test rig has been establishing leading to a change the region of contact between the cylinder liner and the piston compression through a different angle.

The purpose of this work is to demonstrate the effect of cylinder and liners rotation on the overall wear rate by weighting both the cylinder and the liners. The overall worn out in the cylinder of an Automotive Diesel Engine is related to geometrical distortions that lead to the oval cross-section, and no uniform wear rate along the piston-liner cylinder contacting line. Thus, design improvements may be suggested in the light of the analysis of the obtained measurements within the relevant uncertainties. Innovative design modification may lead to extending the engine service life span and minimizing the running operational and maintenance expenses.

## 2 Experimental

**Fig. 1:**
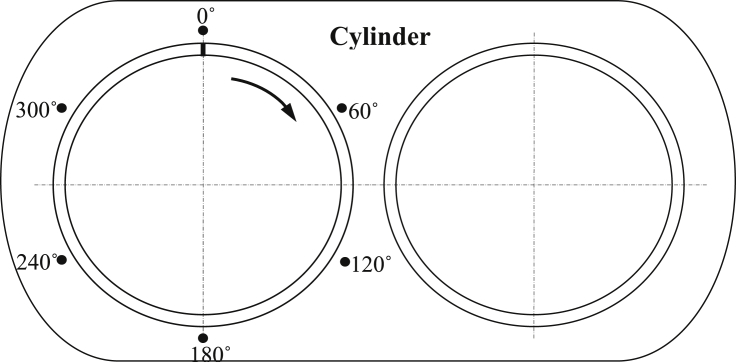
Sketch of the experimental setup.

In order to isolate the effect of thermal stresses on the cylinder liner wear rate, tests with no firing condition (unfired cycle) were carried out. A variable speed electrical motor was used to drive the piston-cylinder system. Fig. 1 shows twin cylinders reciprocating diesel engine that has been used to carry out a series of experiments. One of these pistons is stationary throughout the entire study and the other is rotated once at a specified angle for every driving speed. Tests duration was kept constant that is six hours. Analytical balance of very high precision was used (±0.10 mg) to weight the piston, cylinder and the liners before and after each test.

Fig. 1 shows a sketch of the experimental setup where the rotating speed was measured via tachometer.

**Fig. 2:**
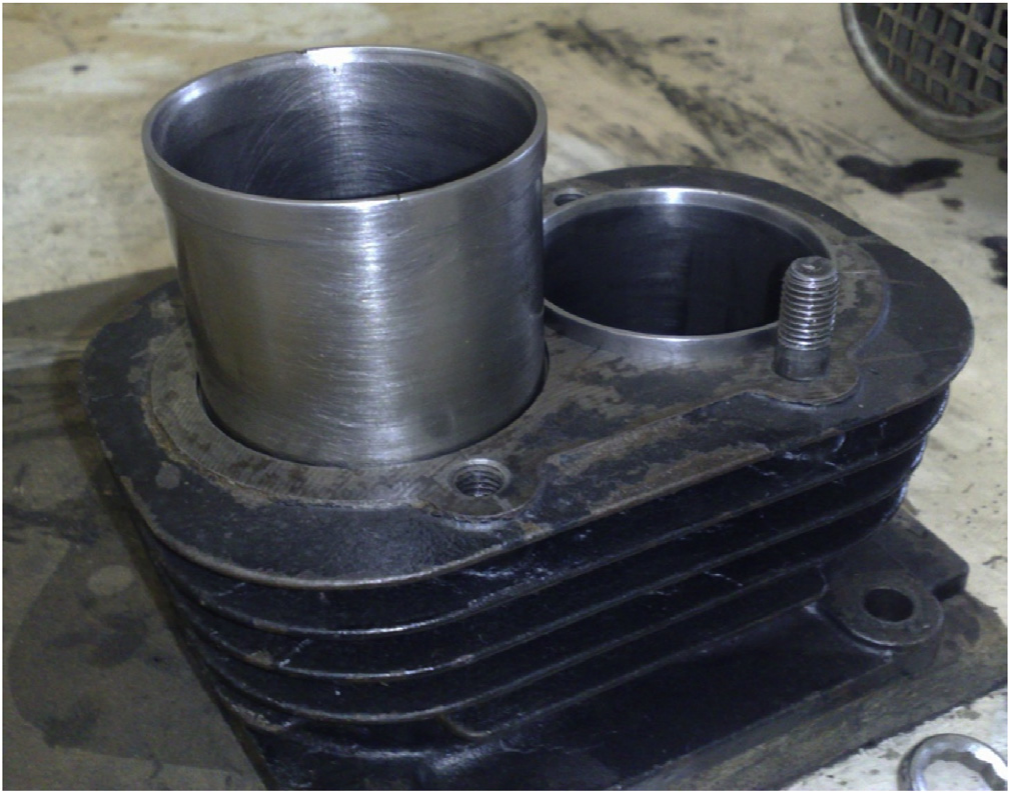
Photograph the fixed and rotating cylinders.

The experiments start after checking the oil level in the engine oil-pan. Then movable cylinder was rotated 60° degrees from its original position besides the fixed and rotating cylinders are depicted in Fig. 2. An approximate constant driving speed was delivered by the electrical motor. A gear-box arrangement of 1000 rpm for the first hour and 3500 rpm for the last hour of the six-hour experiment duration was applied. After each test, cylinder and rings were weighted. The same procedure was followed for the experiments with six different speeds and angels.

## 3 Results & discussion

**Fig. 3:**
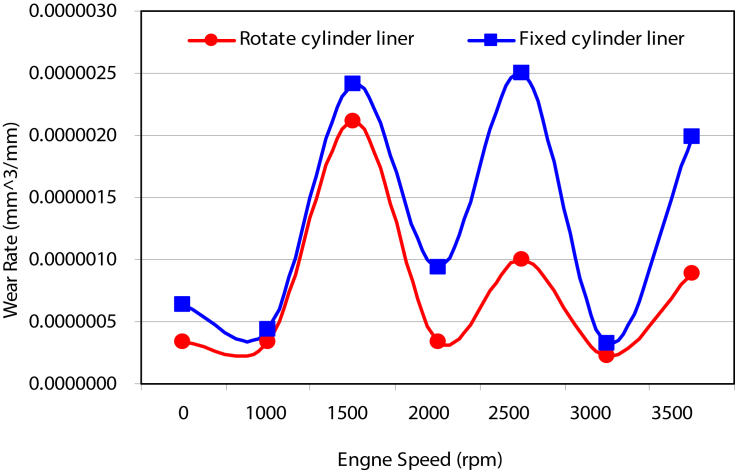
Variation of wear rate with rotating speed.

Fig. 3 shows the variation of wear rate at different values of engine speed. A high wear rate with sinusoidal trend was observed for the case where the stationary cylinder was considered. Whereas, a cyclic trend with dimensioning peeks was observed for the rotated cylinder liner. This difference in behavior is due to changes in the contact surfaces for the case of moving cylinder that leads to decrease in cylinder ovality.

The cyclic trend of wear arises from the vibration mode in journal bearings that supports crankshaft in its rotational motion. An oil film squeezed between the shaft and the outer bearing shell maintain the axis of the crankshaft rotating off-center from that of the bearing center. Vibration modes depend on the engine speed and its harshness reflected on the wear rate curves as the engine speed varies. This affects appears in the results of both sets of experiments denoted by fixed cylinder liner and rotate cylinder liner. Never the less, the wear rate associated with rotate cylinder liner case is lower than that associated with fixed cylinder liner.

The piston-cylinder contacting surface is always the same. However, the non-uniformity of mechanical stresses along piston-cylinder interface increases with continuous increase in cylinder ovality and leads to higher stress levels in localized regions and consequently leads to higher wear rate. Rotating cylinder liner plays an important role in reducing cylinder out of roundness and maintains lower levels of stresses and non-uniformity along the contact interface.

The wear takes place along a contact surfaces inform of longitudinal groves in cylinder liners and burrs in piston rings or vice versa also longitudinal grooves on both cylinder liner and piston rings is possible in the case where quartz grain squeezed between the ring and the cylinder, rotating the cylinder will form a new lines of contact between the two engine parts and displaced the squeezed particles and reduce their effect on wear rate.

**Fig. 4:**
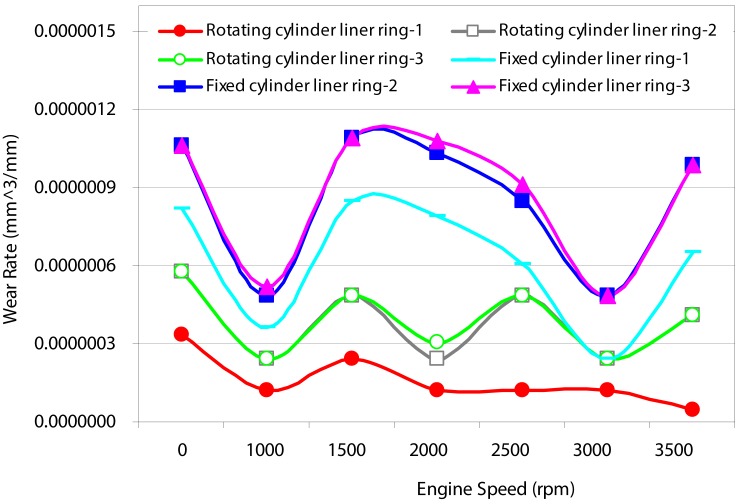
Variation of wear rate with rotating speed.

Fig. 4 shows a comparison of wear rate in various components of the cylinder piston arrangements under consideration. The wear rate associated with the movable cylinder is approximately half in magnitude as compared to the wear rates measured for the stationary cylinder l.

**Fig. 5:**
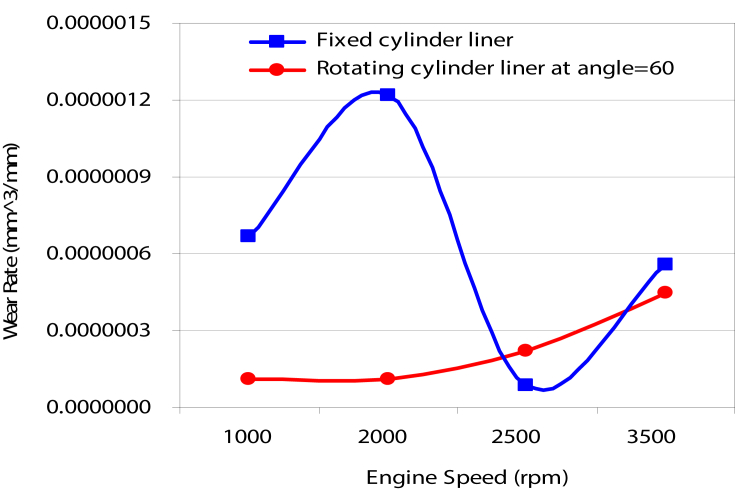
Wear rate of cylinder liner (fixed cylinder liner, rotating cylinder liner positioned at 60^o^angle.

**Fig. 6:**
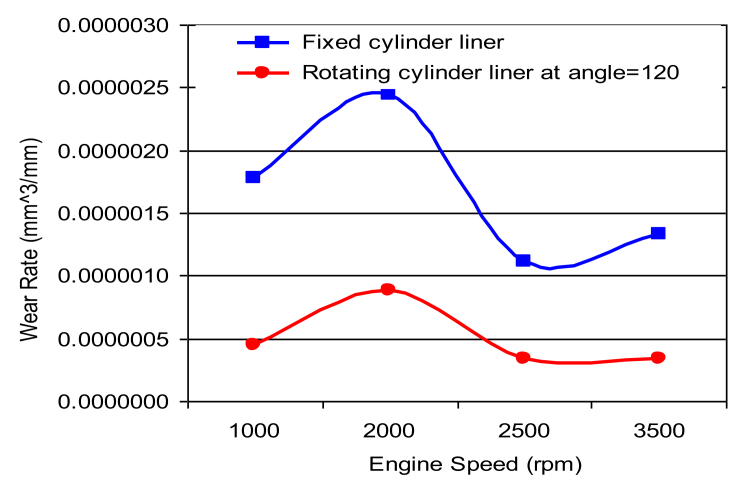
Wear rate of cylinder liner (fixed cylinder liner, rotating cylinder liner positioned at 120^o^ angle.

**Fig. 7:**
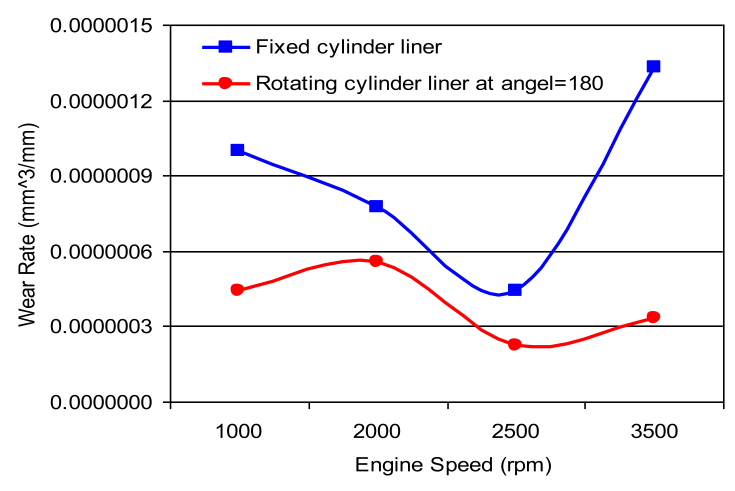
Wear rate of cylinder liner (rotating cylinder liner positioned at 180^o^ angle.

**Fig. 8:**
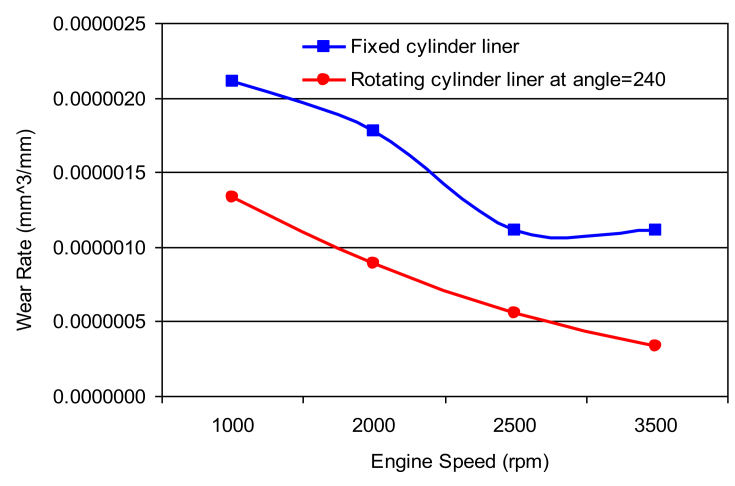
Wear rate of cylinder liner (rotating cylinder liner positioned at 240^o^ angle.

**Fig. 9:**
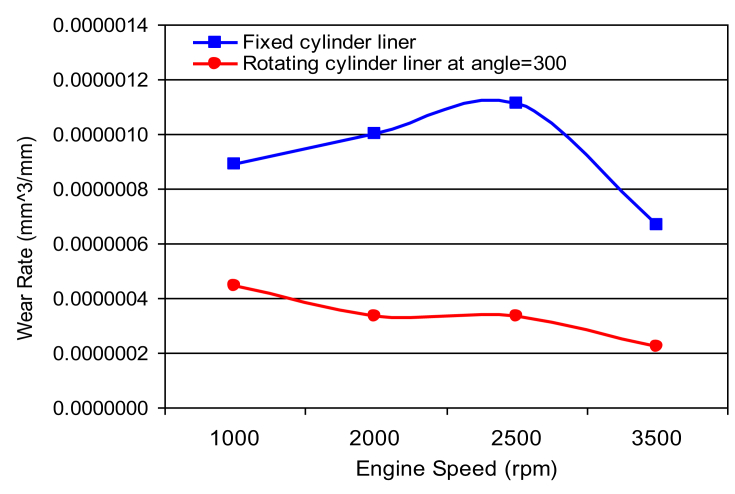
Wear rate of cylinder liner (rotating cylinder liner positioned at 300^o^ angle.

**Fig. 10:**
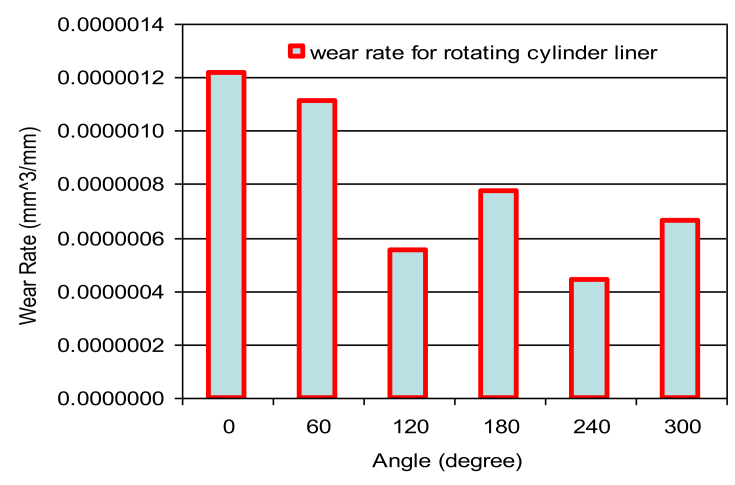
Wear rate of rotating cylinder liner at constant speed (3000 RPM) at different angels.

Figs. 4, 5, 6, 7, 8, 9, and 10 show the variation in wear rate in fixed cylinder liner and rotating cylinder liner at different rotating angles. For movable cylinder liner, angles of 60^o^, 120^o^, 180^o^, 240^o^, and 300^o^ were employed to investigate the effect rotation angle on the wear rate of the piston-rings system. The same operating conditions for two cylinders were maintained for the purpose of comparison. For the case of 60 ^o^ angle of rotation, the comportment of wear rate for different components of piston-cylinder systems at different engine speeds for both scenarios under investigation is shown in Figs. 4, 5. Wear rates in the second and third rings are almost the same in both trend and magnitude for both scenarios (rotating and fixed cylinders) and higher in magnitude than the wear rate in the first ring. A drop in wear rate to almost 50% in the three rings was noticed for the case of rotating cylinder as compared to the wear rate associated with fix cylinder. This is due to the fact that the effect of cylinder ovality decreases as the cylinder rotates consequently the variation of stresses on the contact area between the piston rings and the cylinder surface is small (no localized stresses regions) and less wear takes place. For cylinder liner, reduction in wear rate associated with the 60^o^ angle of rotation is noticeable at low engine speeds specifically less than 2000 rpm for higher engine speeds there is almost no effect on the wear rate.

Figs. 6, 7, 8 and 9 shows the wear rate curves for different rotation angles namely 120^o^, 180^o^, 240^o^, and 300^o^ all figures show the same trend with different magnitudes. The wear rate in cylinder liner associated with rotating cylinder liner is much less than the wear rate associated with fixed liner when compared to the same running conditions and speeds. Fig. 10 shows the variation of wear rate of a rotating cylinder line with the rotational angle at a fixed running speed of 3000 rpm. The general observation was that rotating a cylinder linear has the beneficial effect of reducing the wear rate in all components of the piston-cylinder arrangement. The decrease in wear rate was more obvious in the cylinder liner at rotation angles of 120^o^ and 240^o^ and it is almost one-fourth of the wear that occurs in the stationary cylinder liner.

## 4 Conclusion

A new approach for reducing the wear in internal combustion engines was introduced. It is based on changing the region of contact between the cylinder liner and the piston compression and oil rings. This may be achieved by rotating the cylinder liner every period of time. In the present study, the time duration was six hours. A reduction of an average wear rate to almost half was observed in rotating cylinder as it compared to the stationary cylinder. The effect of engine rotational speed was also investigated. There is not enough evidence that the operating speed has an effect on the difference of wear rate between the two arrangements under investigation (rotating cylinder and fixed cylinder). The current work shows a potential of reducing wear rate in internal combustion engine by simply rotating the cylinder linear by an appropriate angle. However, rotating the cylinder liner while the engine is operating on firing condition is not an easy process and needs a high degree of movement synchronization and control. A drop in wear rate to more than a half was the main feature of rotating cylinder liner.

## Declarations

### Author Contribution Statement

Sa'ed A. Musmar, Ammar Alrousan & I. Tlili: Conceived and designed the experiments; Performed the experiments; Analyzed and interpreted the data; Contributed reagents, materials, analysis tools or data; Wrote the paper.

### Funding Statement

This research did not receive any specific grant from funding agencies in the public, commercial, or not-for-profit sectors.

### Competing Interest Statement

The authors declare no conflict of interest.

### Additional Information

No additional information is available for this paper.

## References

[1] Singh R.C., Lal Roop., Ranganath M., Chaudhary Rajiv (Sept. 2014). Failure of piston in IC engines: a review”. Int. J. Modern Eng. Res. (IJMER).

[2] Vatavuk J., Demarchi V. (1993). Improvement of cylinder liner materials wear resistance. SAE.

[3] Al-Rousan Ammar A. (2006). Effect of dust and sulfur content on the rate of wear of diesel engines working in the Jordanian desert. Alexandria Eng. J..

[4] Bolander N.W., Steenwyk B.D., Ashwin K., Sadeghi F. (2004). Film thickness and friction measurement of piston ring cylinder liner contact with corresponding modeling including mixed lubrication. Conference of the ASME Internal Combustion Engine Division.

[5] Neale M.J. (1995). The Tribology Handbook.

[6] El-Sherbiny M. (1982). Cylinder Liner Wear,9th Leeds-Lion Symposium on Tribology.

[7] Krzyzak Zenon, Pawlus Pawel (2006).

[8] Söderfjäll Markus, Almqvist Andreas, Larsson Roland (2016). Component test for simulation of piston ring – cylinder liner friction at realistic speeds. Tribol. Int..

[9] Biberger Julian, Füßer Hans-Jürgen (2017). Development of a test method for a realistic, single parameter-dependent analysis of piston ring versus cylinder liner contacts with a rotational tribometer’ Tribology. International.

[10] Zhang Zhinan, Liu Zun, Wu Tonghai, Xie Youbai (2017).

[11] Zabalaa B., Igartua A., Fernández X., Priestner C., Ofner H., Knaus O., Abramczuk M., Tribotte P., Girot F., Roman E., Nevshup R. (2017). Friction and wear of a piston ring/cylinder liner at the top dead centre: experimental study and modelling’ Tribology. International.

[12] Zhang Jian, Li Hongwei (2016). Influence of manganese phosphating on’. Surf. Coating. Technol..

[13] Schneider E.W., Blossfeld D.H., Lechman D.C., Hill R.F., Reising R.F., Brevick J.E. (1993). Effect of cylinder bore out-of-roundness on piston ring rotation and engine oil consumption. SAE.

[14] Ohlsson R. (1996). A Topographic Study of Functional Surfaces.

[15] Andersson P., Tamminen J. (2002). Piston Ring Tribology: A Literature Survey.

[16] Jayadas N.H., Nair K.P., Ajithkumar G. (2007). Tribological evaluation of coconut oil as an environment-friendly lubricant. Tribol. Int..

[17] Nabnu T., Ren N., Yasuda Y., Zhu D., Wang Q.J. (2008). Micro-textures in concentrated conformal-contact lubrication: effects of texture bottom shape and surface relative motion. Tribol. Lett..

[18] Meng Xianghui, Hu Yang, Xie Youbai (2016). Modeling of the cylinder Liner “Zero-wear” Process by Two-Scale Homogenization Technique’ Wear 368-369.

[19] Spiller S., Lenauer C., Wopelka T., Jech M. (2017). Real time durability of tribofilms in the piston ring – cylinder liner contact’ Tribology. International.

[20] Jayakumar N., Mohanamurugan S., Rajavel R. (2017). Study of wear in chrome plated cylinder liner in two stroke marine diesel engine lubricated by hans jensen swirl injection. Prin. Mater. Today Proceed..

[21] Rahmani R., Rahnejat H., Fitzsimons B., Dowson D. (2017). The effect of cylinder liner operating temperature on frictional loss and engine emissions in piston ring conjunction’. Appl. Energy.

